# Plasma Exosomal miRNAs in Persons with and without Alzheimer Disease: Altered Expression and Prospects for Biomarkers

**DOI:** 10.1371/journal.pone.0139233

**Published:** 2015-10-01

**Authors:** Giovanni Lugli, Aaron M. Cohen, David A. Bennett, Raj C. Shah, Christopher J. Fields, Alvaro G. Hernandez, Neil R. Smalheiser

**Affiliations:** 1 Department of Pathology, University of Illinois at Chicago, Chicago, Illinois, United States of America; 2 Department of Medical Informatics and Clinical Epidemiology, Oregon Health & Science University, Portland, Oregon, United States of America; 3 Rush Alzheimer’s Disease Center, Rush University, Chicago, Illinois, United States of America; 4 HPCBio, University of Illinois, Urbana, Illinois, United States of America; 5 Roy J. Carver Biotechnology Center, Urbana, Illinois, United States of America; 6 Department of Psychiatry and Psychiatric Institute, University of Illinois at Chicago, Chicago, Illinois, United States of America; East Carolina University, UNITED STATES

## Abstract

To assess the value of exosomal miRNAs as biomarkers for Alzheimer disease (AD), the expression of microRNAs was measured in a plasma fraction enriched in exosomes by differential centrifugation, using Illumina deep sequencing. Samples from 35 persons with a clinical diagnosis of AD dementia were compared to 35 age and sex matched controls. Although these samples contained less than 0.1 microgram of total RNA, deep sequencing gave reliable and informative results. Twenty miRNAs showed significant differences in the AD group in initial screening (miR-23b-3p, miR-24-3p, miR-29b-3p, miR-125b-5p, miR-138-5p, miR-139-5p, miR-141-3p, miR-150-5p, miR-152-3p, miR-185-5p, miR-338-3p, miR-342-3p, miR-342-5p, miR-548at-5p, miR-659-5p, miR-3065-5p, miR-3613-3p, miR-3916, miR-4772-3p, miR-5001-3p), many of which satisfied additional biological and statistical criteria, and among which a panel of seven miRNAs were highly informative in a machine learning model for predicting AD status of individual samples with 83–89% accuracy. This performance is not due to over-fitting, because a) we used separate samples for training and testing, and b) similar performance was achieved when tested on technical replicate data. Perhaps the most interesting single miRNA was miR-342-3p, which was a) expressed in the AD group at about 60% of control levels, b) highly correlated with several of the other miRNAs that were significantly down-regulated in AD, and c) was also reported to be down-regulated in AD in two previous studies. The findings warrant replication and follow-up with a larger cohort of patients and controls who have been carefully characterized in terms of cognitive and imaging data, other biomarkers (e.g., CSF amyloid and tau levels) and risk factors (e.g., apoE4 status), and who are sampled repeatedly over time. Integrating miRNA expression data with other data is likely to provide informative and robust biomarkers in Alzheimer disease.

## Introduction

Because Alzheimer disease (AD) is chronic, progressive, and increasingly prevalent, and has a long asymptomatic latency period, many investigators are searching for biomarkers that can detect the disease as well as monitor its course, particularly in its pre-symptomatic and early stages [1, 2], which may allow earlier treatment [3] and disease management to be initiated. Approaches to biomarkers include neuro-imaging parameters, and individual proteins or proteomic profiles in CSF, blood and plasma or serum. Recently, several studies have reported changes in levels of microRNAs (miRNAs) as measured in postmortem brain studies [4–9], as well as miRNA changes detected in whole blood, plasma, or serum [10–24].

Although the RNA biomarker approach is promising, it is not yet clear which tissue source or type of RNAs will give optimal biological information and optimal predictive value in a clinical setting. We hypothesized that isolated circulating exosomes might be a particularly “clean” and informative tissue source, since they are secreted physiologically by many tissues and the secretion can be actively modulated in a tissue-specific manner. There is suggestive evidence that the pool of circulating miRNAs contains miRNAs released from neural tissue (either from CNS, peripheral or autonomic neurons) [16, 25–29]. Release of neuronal exosomes is modulated by synaptic activity and release sites can be localized in dendrites, so pathophysiological alterations in AD might be reflected in the number or composition of neuronal exosomes. Although neuronal exosomes are likely to represent a small fraction of overall exosomes in the circulation, they may be detectable via monitoring miRNAs that are highly enriched in brain, and might be isolated via immune-adsorption with antibodies such as L1CAM [29].

There is a potential problem in studying highly purified circulating exosomes in clinical samples, namely, that the yield of purified exosomes in serum or plasma is rather small. Exosomes present in 1 ml of plasma contain less than 0.1 microgram of total RNA, whereas deep sequencing protocols have routinely required 1 microgram or more total RNA. Several studies dealt with this issue by studying “exosomal RNA” isolated from serum or plasma via commercial kits. These kits provide comprehensive isolation of exosomes but have extremely high yields, and to our knowledge, these kit-based methods have not been shown to purify exosomes away from more abundant but biologically distinct RNA-containing fractions (e.g. microvesicles or soluble protein complexes). To our knowledge, no studies have carried out deep sequencing analyses on highly purified plasma exosomes, and so it was not known whether the RNA yield and quality would be sufficient and consistent for deep sequencing purposes.

In the present paper, we measured the expression of microRNAs and other small RNAs in a plasma fraction enriched in exosomes by differential centrifugation, using the Illumina deep sequencing method, comparing expression in persons with a clinical diagnosis of Alzheimer disease dementia vs. age and sex matched controls. (Thus, we looked for biomarkers that correlate with diagnosis rather than attempting to predict which individuals would exhibit dementia at a future date.) We validated the methodology for measuring circulating exosomes in clinical samples by deep sequencing. The AD group exhibited a number of informative miRNA changes, and a machine learning model was able to assign individual samples to the AD vs. control group with relatively high accuracy.

## Materials and Methods

### Recruitment of participants

Participants were recruited from the Rush Memory Clinic Data Repository (Rush University, Chicago, IL, USA). The participant selection flow diagram is shown in S1 Fig. Persons with dementia due to Alzheimer’s disease and matched healthy controls were recruited from clinic and community sites. The main source of persons with dementia due to Alzheimer’s disease was the clinic (n = 46, 92%) and the main source of healthy controls (n = 41, 82%) were from the community.

Clinic participants presented to the Rush Memory Clinic, a specialty referral clinic at the Rush University Medical Center, for evaluation of their cognition, as previously described [30, 31]. Clinic participants underwent uniform, structured, clinical evaluations. As previously reported [32], evaluations included a detailed medical history, neurologic examination, cognitive function testing, brief psychiatric evaluation, and an interview with a knowledgeable informant. Ancillary tests (e.g., laboratory testing, structural neuroimaging, examination of cerebrospinal fluid, positron emission tomography) were obtained when clinically indicated. The procedures were compatible with the Consortium to Establish a Registry for Alzheimer’s Disease (CERAD) [33], similar to those conducted by the Clinical Cores of other federally-funded AD Centers, and consistent with the current practice parameters for the diagnostic evaluation for dementia [34]. Participants with normal memory function deemed by an experienced clinician after the evaluation (n = 9) were included in the healthy control group.

Community participants were recruited from community-based studies, support groups, and presentations. Community participants mainly were spouses of persons diagnosed with dementia due to Alzheimer’s disease. As part of the Rush Memory Clinic Data Repository, the community participants without dementia (n = 41) had a recorded medical history which included self-report about not being diagnosed with dementia. The majority (n = 32) also had screening cognitive testing obtained in person or via the telephone. Community participants diagnosed with dementia due to Alzheimer’s disease (n = 4) had medical records requested for review regarding the diagnosis.

For this study, inclusion criteria included being a man and/or woman between ages 50 and 75 inclusive and having either dementia due to Alzheimer’s disease or no dementia. Exclusion criteria: people on heparin therapy at the time of blood draw (since heparin can interfere with RNA isolation), as well as people who were diagnosed with any non-Alzheimer type of dementia.

### Ethics

The Rush Memory Clinic Data and Specimen Repository is a project approved by the Rush University Medical Center Institutional Review Board. At the time of the initial visit, the patient was presented with information regarding enrollment in the Rush Memory Clinic Data and Specimen Repository. An informed written consent approved by the Rush University Medical Center Institutional Review Board for this study was voluntarily signed by the patient and/or the appropriate surrogate if the patient did not have the capacity to consent. Samples and de-identified data from the Repository were provided to the investigators based on a process approved by the Rush University Medical Center Institutional Review Board. The UIC Office for the Protection of Research Subjects determined that the RNA expression measurements conducted at UIC in this study (study of de-identified samples obtained from the Rush Repository) does not meet the definition of human subject research as defined by 45 CFR 46.102(f).

### Collection of plasma

Samples of platelet-poor plasma were obtained (using lavender top EDTA tubes) and stored at the Rush Alzheimer Disease Center Repository. Controls were matched individually with patient samples for age (within a range of 5 years), gender, and plasma storage time. Most blood draws also collected aliquots for CBC and differential data. Initially, we carried out methodological tests on 10 samples (5 in each group) that had been drawn ~5 years earlier, and then examined a larger cohort of 76 samples that had been drawn less than 1 year prior to RNA isolation and subsequently stored at -80C. To reduce group heterogeneity in this initial study, all samples were obtained from subjects of European descent. Of the 76 samples collected and processed, 71 formed adequate cDNA libraries and were sequenced. In one sample, which was marked during processing as possibly having lost the RNA pellet, sequencing was performed but its small RNA expression was anomalously low, so it was excluded from analysis, leaving a total of 70 samples (35 in each group) which were analyzed for the results reported here (see demographics in S1 Table). All analyses were done under blind conditions, i.e. the investigators who prepared the samples and those who carried out the data analyses were unaware of group identity. Samples were marked “group A” and “group B” so that an equal number of samples in each group were processed together during RNA isolation and RNA sequencing.

### Exosomal enrichment

Initial methodological studies of exosomal enrichment, RNA isolation and characterization were carried out using commercially obtained normal adult mouse and human plasma (mouse plasma from Equitech-Bio, INC., Kerrville, TX, Lot # MPE07-483; human plasma from Bioreclamation, LLC., Westbury, NY, Lot # BRH611575). To verify that our methods could reliably detect miRNA expression in an exosomal fraction of human plasma, and to optimize the preparative and analytical methods prior to preparing the clinical samples analyzed in this study, we first processed ten archival samples of human plasma (five in each group) obtained from the Rush Alzheimer Disease Center Repository and carried out deep sequencing.

**Fig 1:**
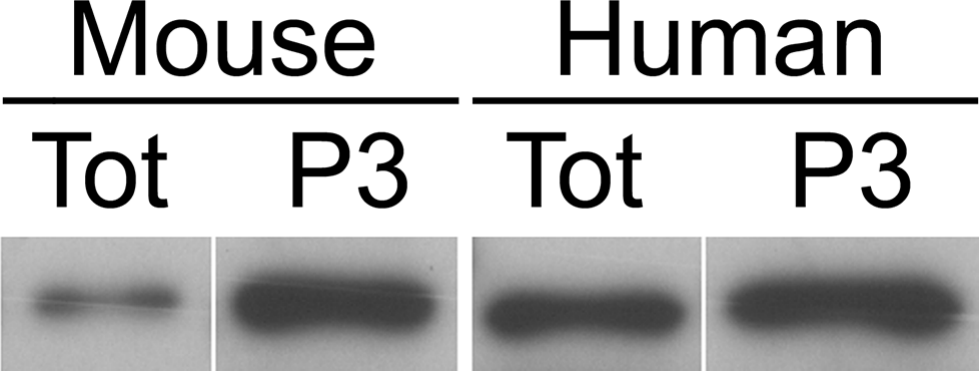
Expression of Alix in whole plasma vs. P3 fraction. Fractions were prepared from normal mouse and human plasma, and equal amounts of protein were loaded for immunoblotting using anti-Alix antibody (see Methods). The P3 fractions were positive for Alix and were enriched relative to whole plasma.

The exosomal-enriched fraction of plasma was obtained by thawing 4 ml of frozen plasma (thawing was carried out at 4C); a cocktail of protease and RNase inhibitors were added in a volume of 70 μL/mL to reach the final concentration desired (10 mM EDTA, 10mM NEM, 1 mM PMSF, 1 μg/mL Pepstatin A, 10 μg/mL Leupeptin, 2 μg/mL Aprotinin, 10mM NaF, 1mM Na_3_VO_4_, 160 U/mL SuperaseIN (Life Technologies, Grand Island, NY), 160 U/mL RNase OUT, (Life Technologies). Samples were spun down at 1,500g x 10 min at 4C; the S1 supernatant was collected and diluted 1:3 with RNase free PBS and spun down at 20,000xg for 20 min. The dilution was performed to improve yield and to minimize variability in osmolarity that might be associated with clinical samples. The S2 supernatant was collected and further spun down at 200,000xg for 2 h at 4C using a 41Ti rotor. The final P3 pellet was used as the exosomal-enriched fraction. The P3 fraction was enriched in Alix, a marker of exosomes, relative to total plasma (Fig 1). Although the total amount of RNA present in a pellet was too small to allow accurate quantification by the nanodrop method, plasma miRNAs such as let-7a and mir-16 were readily measured by manual RT-qPCR (see below).

### RNA isolation

1 mL of Trizol (Life Technologies) was added to the P3 pellet with 20 μL of spike-in synthetic RNA (see below). Glycoblue (Life Technologies) (22.5 μg) and isopropanol (600 μl) were added to the aqueous phase, and incubated overnight at -20C. Samples were spun down at 20,000xg for 30 min, rinsed once with 80% EtOH (1.4 mL), and air-dried. The RNA was then re-suspended in 35 μL of RNAsecure (Life Technologies), heated at 60C for 20 min, then DNase I treatment was performed with Turbo-DNA free kit (Life Technologies) following manufacturer’s instructions. 40 μL final volume per sample was collected and stored at -80C.

### Exogenous spike-in synthetic RNA

Synthetic ath-miR-159a was bought from IDT (Coralville, IA) and utilized as an exogenous spike-in control RNA which is not expressed in mammalian species. The RNA was re-suspended to 200 μM with TE buffer and then further diluted to obtain a working stock concentration of 0.655 pM in TE buffer. It was aliquoted in single working solutions and stored at -20C. A 20 μL aliquot (at 0.655 pM) was added directly to Trizol at the time that the P3 pellet was solubilized.

### Characterization of samples prior to deep sequencing

Levels of ath-mir-159a, hsa-mir-16 and hsa-let-7a were measured by manual RT-PCR in the first 31 samples to ensure that the yield and quality of the RNA was consistent and adequate in all cases. The RT-qPCR for these RNAs was performed as previously described [35] using 5 μL of RNA. A standard curve of the spiked-in control RNA was examined to assess its recovery and linearity. Pilot studies suggested that adding more glycogen carrier (160 μg/1mL Trizol) directly to Trizol, instead of to the aqueous phase, would improve RNA yield. This change was implemented for samples #25 and later. This modification increased the number of raw sequence counts and mappable reads by an average of ~23%, but should not have altered the results reported here since a) the modification affected equal numbers of samples in the AD and control groups, and b) raw sequence counts were normalized by the total number of genome mappable reads.

### Western Blotting

An aliquot of the whole plasma and of the P3 fraction were methanol precipitated overnight at -20C, spun down at 20,000xg for 20 min and air dried overnight under the hood. Protein concentration was determined by Lowry assay. The dry-pellets were resuspended in SDS-PAGE sample buffer containing 1% DTT, adjusted to contain equal amounts of protein, and boiled (5 min) prior to loading. Samples were separated on 4–15% SDS-PAGE Criterion gels (Bio-Rad, Hercules, CA) run in parallel with Bio-Rad Precision Plus-Protein dual color standards molecular-weight (Bio-Rad, Hercules, CA), and transferred to Hybond-P PVDF membranes (Amersham, Piscataway, NJ). Blots were blocked in 1% non-fat dry milk in PBS for 1 h at room temperature, incubated with primary polyclonal antibody anti-Alix (Cat# ABC40, lot # 1984695, EMD Millipore, Billerica, MA, USA) overnight with rocking at 4°C and rinsed. Goat anti-rabbit IgG (peroxidase conjugated; Sigma A0545, sigma, St Louis, MO, USA) was added at 1:30,000 for 2 h and rinsed. Finally, blots were incubated in ECL-Plus reagent (Amersham, Piscataway, NJ) and exposed to film (Hyperfilm ECL, Amersham, Piscataway, NJ). As negative controls, primary antibody was omitted or irrelevant primary antibodies were used.

### Deep Sequencing Methods

#### Construction of small RNA libraries

Construction of libraries and sequencing on the Illumina HiSeq2500 were performed at the W. M. Keck Center for Comparative and Functional Genomics at the University of Illinois at Urbana-Champaign. Small RNA libraries were constructed from the RNA samples using the TruSeq Small RNA Sample Preparation Kit (Illumina, CA) with two modifications: the final libraries were amplified by PCR for 16 cycles with the Kapa HiFi polymerase (Kapa Biosystems, MA), and the individually-barcoded libraries were mixed into 7 pools and the pools were size selected on Novex 10% TBE gels (Life Technologies) to enrich for small RNAs 18 to 50nt in length. Barcoding involved 8–12 samples sequenced per lane, and was balanced so that equal numbers of samples from group A and B were processed and sequenced in parallel. The final libraries were quantitated by Qubit (Life Technologies) and the average size was determined on an Agilent Bioanalyzer High Sensitivity DNA chip (Agilent Technologies, DE) and diluted to 10 nM. The pooled libraries were further quantitated by qPCR on an ABI 7900 (Life Technologies).

#### Sequencing on an Illumina HiSeq2500

Each pool was loaded onto two lanes of an 8-lane flowcell for cluster formation and sequenced on an Illumina HiSeq2500. One of the lanes in each flowcell was loaded with a PhiX Control library that provides a balanced genome for calculation of matrix, phasing and prephasing. The libraries were sequenced from one end of the molecules to a total read length of 50nt. The raw.bcl files were converted into demultiplexed fastq files with Casava 1.8.2 (Illumina).

#### Bioinformatics cleansing and preparation of sequences for analysis

Raw sequence counts were processed in a bioinformatics pipeline including a quality control filter, trimming of adapters, consolidation of reads across samples, and alignment of reads against reference databases. The details are described in S2 Fig and S1 Methods.

#### Normalization of raw sequence counts

Non-normalized data and several normalization schemes were examined to learn which method gave best results, in terms of lowest expression variability within groups and most sensitive detection of differences across groups. Counts normalized per million mappable reads to human genome gave better results than counts normalized per geometric mean of the exogenous spike-in control RNA (ath-mir-159a) and an endogenous RNA (hsa-mir-486-5p, one of the most abundant miRNAs, which showed no mean change and low variability across groups). We noted that mir-16, which is popular in other studies as a normalizing factor, was not suitable in our study since it did not have equal mean expression across groups.

### Data analysis

#### Expression analysis and statistics

The dataset was filtered to keep sequence reads that showed at least a total (across the 70 samples) of 500 raw sequence counts before normalization. Each sequence read that aligned to the mature miRNA database was normalized and tabulated in terms of its expression in each sample, its overall sum, its mean expression in each group, the ratio across groups, and measures of statistical significance using a variety of tests: a) two-tailed t-test with unequal variance; b) one-way ANOVA, c) ANOVA blocked by RNA preparation date; d) Kruskal-Wallis test (nonparametric ANOVA); and e) nonparametric Wilcoxon test. This is attached as S1 Dataset (note that only t-test and Kruskal-Wallis tests are displayed in this file). In most cases, the different statistical tests gave similar results. Because the data do not necessarily follow a normal distribution, the Kruskal-Wallis test was chosen for initial screening to identify sequence reads that showed differences among groups at nominal significance at p = 0.05 or better (Table 1). Sequences were then analyzed further as described in Results.

**Table 1:** miRNAs showing differential expression in this study.

miRNA	AD mean	Control mean	AD/Control	Kruskal test	t test	5'seed	most abundant expressed seq
miR-185-5p	24.10	44.08	0.546	0.0012	0.0410	GGAGAGA	TGGAGAG**AAAG**GCAGTTCCTG
**miR-342-3p**	340.63	547.94	0.621	0.0039	0.0007	C**TCACA**C	TCTCACACAGAAATCGCACCCGT
**miR-141-3p**	55.43	96.00	0.577	0.0044	0.0109	AACACTG	TAACACTGTCTGGT**AAAG**ATG
miR-548at-5p	2.37	0.73	3.238	0.0129	0.0051	AAAGTTA	A**AAAG**TTATTGCGGTTTTGGCT
**miR-342-5p**	138.24	196.86	0.702	0.0197	0.0078	GGGGTGC	AGGGGTGCTATCTGTGATTGA
miR-4772-3p	5.44	10.96	0.496	0.0240	0.0398	CTGCAAC	CCTGCAACTTTGCCTGATCAGA
**miR-23b-3p**	285.73	378.79	0.754	0.0252	0.0228	**TCACA**TT	ATCACATTGCCAGGGAT
miR-138-5p	14.63	12.42	1.177	0.0304	0.7101	GCTGGTG	AGCTGGTGTTGTGAATCAGGCCG
**miR-24-3p**	21.68	33.89	0.639	0.0311	0.0283	GGCTCAG	TGGCTCAGTTCAGC**AGGA**ACA
miR-29b-3p	35.20	51.61	0.682	0.0330	0.0148	TAGCACC	CTAGCACCATTTGAAATCAGTG
miR-3916	1.27	5.21	0.244	0.0343	0.0489	AAATAGC	GAAATAGCTGGTTCTC
**miR-125b-5p**	108.45	147.98	0.732	0.0370	0.0420	CCCTGAG	T**CCCT**GAGA**CCCT**AACTTGTGA
miR-338-3p	27.08	41.49	0.652	0.0398	0.0178	**CCAGCAT**	TCCAGCATCAGTGATTTTGTT
miR-3065-5p	49.85	72.52	0.687	0.0403	0.0724	**CCAGCAT**	TCCAGCATCAGTGATTTTGTTG
miR-139-5p	36.41	61.22	0.594	0.0415	0.0404	CTACAGT	TCTACAGTGCACGTGTCTCCAGT
**miR-152-3p**	50.16	76.97	0.651	0.0427	0.0629	CAGTGCA	TCAGTGCATGACAGAACTTGGGA
miR-150-5p	3809.19	5076.62	0.750	0.0439	0.0489	CTCCCAA	TCTCCCAA**CCCT**TGTACCAGTG
miR-5001-3p	4.62	1.56	2.954	0.0440	0.0978	TCTGCCT	TTCTGCCTCTGTCCAGGTCCT
miR-659-5p	5.12	1.74	2.939	0.0486	0.0805	GGACCTT	**AGGA**CCTT**CCCT**GAACCA**AGGA**
miR-3613-3p	0.87	2.16	0.406	0.0492	0.0340	CAAAAAA	ACAAAAAAA**AAAG**CCCAA**CCCT**

Shown are miRNAs for whom the sum of all sequences aligning to a given mature miRNA reference sequence (in miRBAse) is significantly different across groups at p = 0.05 or better by Kruskal-Wallis test. In bold are miRNAs whose most abundant expressed sequence is also significantly different across groups. (Note 6 miRNAs, whose mean expression is less than 10 counts, only expressed a single miRNA sequence mapping to its locus and are not bolded.) Underlined are the 7 miRNAs which were selected in machine learning experiments as most predictive for group identity.

#### Correlation analysis

For each of the 465 miRNA loci to which sequences aligned in the mature miRNA dataset, we tabulated the sum of all sequences aligning to the locus, and calculated the pairwise correlation for all miRNA pairs, both within each group and across the 70 samples as a whole. For this analysis, we used the rank Spearman rho correlation coefficient, rather than the Pearson r, as being more robust. We also calculated the p-value for each pair, giving the confidence that the rho value is significantly different from zero. For 35 samples in each group, a rho value of ~0.3 generally is significantly different from zero at p = 0.05. The spreadsheet containing all pairwise correlations is attached as S2 Dataset. As discussed in Results, several different situations were examined, including cliques of miRNAs which were all pairwise highly correlated (rho>0.5) in both groups, as well as those which were highly correlated in one group but showed a much lower correlation in the other (i.e., difference in rho values > 0.5).

#### Machine learning analyses

A machine learning model was built using the mature miRNA expression data in order to predict whether an individual sample belonged to the AD group or control group. The input data consisted of the normalized miRNA expression data (summed for all sequences aligning to each miRNA locus) across 465 loci. These data were converted to the log of the fold changes (AD/control) in the following manner. First the grand mean for each locus was computed across all 70 samples. Then, for each sample, the fold change was computed by dividing the sample expression level by the grand mean for that locus. Fold changes less than 10% (i.e, ratios between 0.90 and 1.10) were rounded to 1.0. Finally, the log base 3 of the fold change was computed, and range limited, resulting in values that lay between -1.0 and +1.0 for all loci for all samples. These values were used as features in classifier feature selection, training, and testing.

During the feature selection process, cross-validation was employed to evaluate the impact of including or excluding values from specific loci as features. Since there were 70 training samples in the initial data set, 35 positive for AD and 35 negative (i.e., controls), 7-way cross-validation was used (i.e., 10 samples per partition). The 70 samples were randomly divided into 7 partitions of 5 positive and 5 negative samples each and cross-validation was performed on these partitions, using 6 partitions for training and one for evaluation at a time. The random partitioning was repeated ten times in order to have 70 estimates of the performance measures of interest. These estimates were averaged in order to access the relative performance of one set of features vs. another according to three standard, commonly used evaluation measures: AUC, F1, and MCC (defined below).

Each machine learning run results in predictions which are either true positive (TP), true negative (TN), false positive (FP), or false negative (FN). Precision = TP / (TP + FP) and recall = TP / (TP + FN). Furthermore, predictions are given a confidence score between 0 and 1, which is used in non-binary rank based measures such as AUC. AUC = area under the receiver operating curve = area under the true positive rate vs. false positive rate curve. F1 = Balanced F-measure, the harmonic mean of precision and recall = 2 * precision * recall / (precision + recall). MCC = Matthew's Correlation Coefficient, a "single number" summary of a confusion matrix of true positive, false positive, true negative, and false negative results.

$$MCC\;=\;\frac{TP\times TN-FP\times FN}{\sqrt{\left(TP+FP)(TP+FN)(TN+FP)(TN+FN\right)}}$$

Three classifier algorithms were evaluated: J48 decision trees (Weka 3.6.11 implementation), SVM (SVMLight V6.02 implementation), and adaboostM1 (Weka 3.6.11). The three algorithms were chosen because they all generally perform well and they use very different mathematical formulations in their approach. Initial experiments using all 465 loci, using all three classifiers, gave poor results with AUC values between 0.60–0.70, probably because the classification algorithms were misled by the large number of irrelevant and potentially redundant features. Clearly some type of feature selection or pruning was necessary. Next, we selected the 50 most significant features, according to the Mann-Whitney U test, as a starting set. Based on these 50 features, the J48 classifier produced the best cross-validation results as input, producing a mean AUC = 0.690, MCC = 0.336, and F1 = 0.641. This is not exceptional performance, but it is certainly much better than random. Based on these initial tests, the J48 decision tree classifier was applied to provide a feature selection method. Using cross-validation as described above, we scored the number of times that a given miRNA locus feature was used as a node in a decision tree for the 70 cross-validation runs. This scoring metric allowed us to rank the discrimination importance of each feature. Counts ranged from 88 (a miRNA could be used at more than one node in a single tree) to 1 for these 50 features. Features were ranked according to their tree counts, and the top scoring 18 features (i.e., miRNA loci) were chosen to move onto the next step.

Several classifiers were evaluated using these 18 features, and adaboostM1 performed the best. AdaboostM1 was therefore chosen for the final feature selection optimization and model construction. The final feature set was chosen using both forward and backward selection with the 18 features. Forward selection starts with a model containing no features, and runs cross-validation on the current model plus each available feature one at a time, in order to determine which feature achieves the greatest improvement in the model. This is then repeated using the new improved model and testing the remaining features until there is no additional feature that improves the model. Backward selection starts with all 18 features in the model, and removes each feature one at a time to determine whether removing a feature from the model results in improved performance during cross-validation. If removing a feature improves performance, then the model is updated to leave out the feature whose removal improves performance the most. The process is then repeated to try removing additional features one by one. The process stops when there is no feature whose removal improves performance. These forward and backward selection processes were performed on the initial dataset and 18 chosen features. At each step, a feature was chosen based on an improvement on AUC or MCC measures. Both forward and backward selection resulted in the same model, consisting of seven miRNA features (i.e., miRNA loci) shown in Table 2.

**Table 2:** Cross-validation performance using the optimized seven miRNA features with different machine learning algorithms.

Method		Precision	Recall	F1	AUC	MCC
AdaboostM1		0.888	0.817	0.836	0.919	0.71
J48		0.784	0.677	0.691	0.747	0.46
SVMLight Linear		0.821	0.723	0.749	0.833	0.57

Shown is the mean performance for each machine learning method (see Methods for details).

The adaboostM1 algorithm was then trained using these seven features with all 70 data samples to create the optimized final model. To validate the selection of adaboostM1 as the algorithm for our final model, cross-validation was also evaluated using J48 decision trees and SVM with the optimized seven miRNA features. AdaboostM1 clearly performed the best with these seven features (Table 2). We also investigated the predictive value of adding features based on modules of miRNAs that were all highly correlated with each other within one group, but not correlated at all with each other in the other group (see Results). However, this did not improve overall performance (data not shown); therefore, the final model was created by training adaboostM1 on the entire 70 sample initial data set using the optimized 7 individual miRNA features.

## Results

### Global assessment of the dataset as a whole

**Fig 2:**
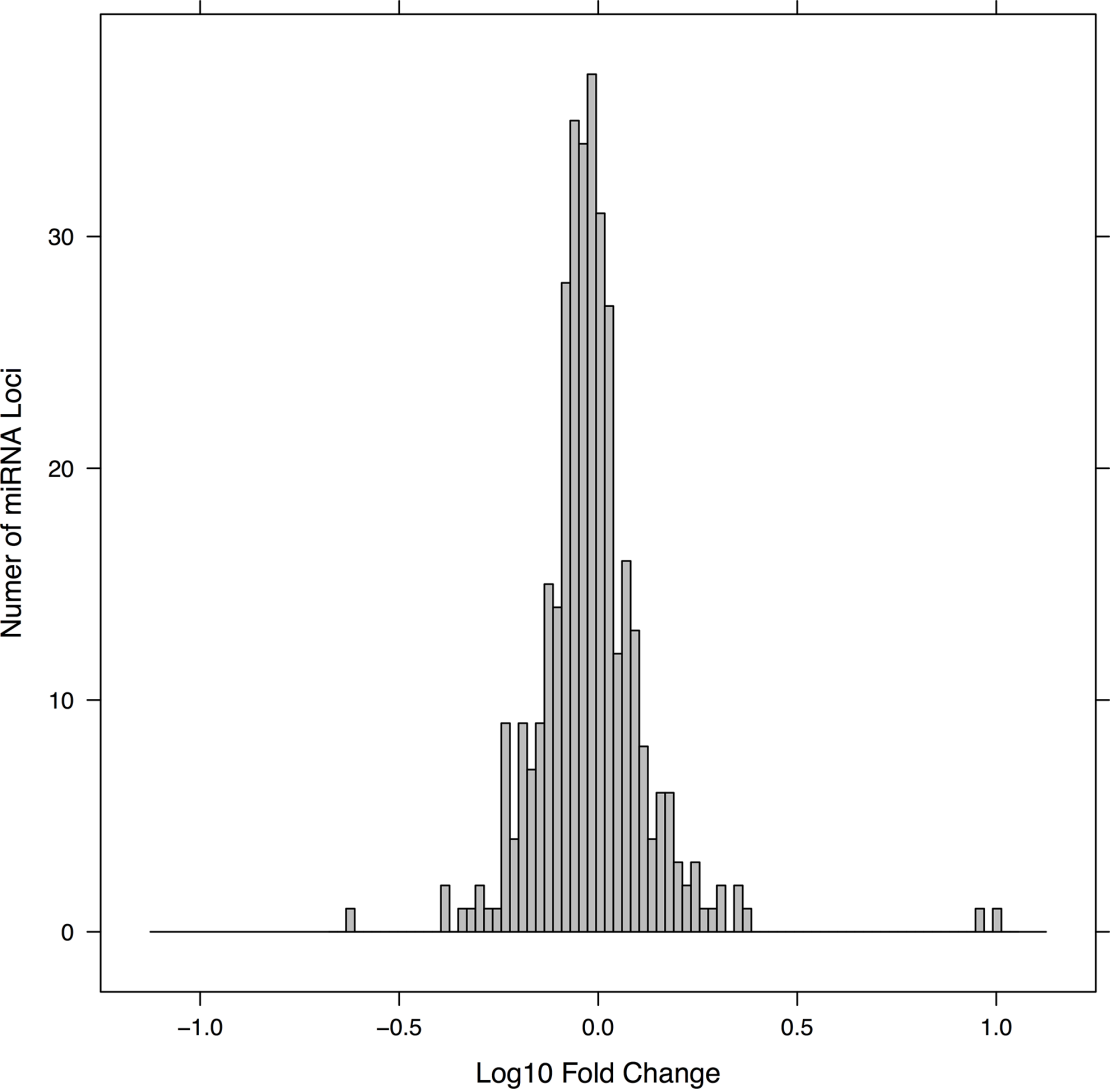
Distribution of fold-changes across mature miRNA loci. This figure shows the mean fold-change (i.e., the AD/control ratio) for mature miRNA loci in the filtered dataset (all sequences aligning to a given locus were summed up to give one value per locus). Shown are only those miRNAs which expressed mean counts of at least 3.6 in the control group (this threshold removes the lowest 25% of loci; low expressing miRNAs were removed to reduce noise and ensure that the ratios are robust). The distribution roughly follows a normal curve, with approximately equal numbers of miRNAs up and down across groups, and most miRNAs showing fold-changes of 2-fold or less. Note that the fold-change is displayed on a log scale (e.g., a value of 1 represents a 10-fold increase and a value of -1 represents a 10-fold decrease).

Overall quality control measures of the mature miRNA expression dataset were similar to previous miRNA expression experiments using recommended amounts of total RNA (i.e., 1–2 micrograms) of fresh brain tissue [36, 37], i.e. in terms of the depth of reads, percentage of raw reads that aligned to the human genome, and the percentage of genome-mappable reads that aligned to miRBase reference mature miRNA or hairpin precursor sequences. The AD and control groups showed similar global read statistics with regard to the total number of raw sequence counts, reads that map to the Hg19 reference human genome, and reads mapping to miRBAse mature or hairpin sequences (S1 Table). After normalizing the raw miRNA sequence counts in each sample relative to the number of human genome mappable reads, there was no global change in expression across the entire population of miRNAs as a whole. As shown in Fig 2, the distribution of mean fold-changes for individual miRNA sequences between AD and control groups is symmetrical and centered at zero, with 95% confidence intervals corresponding to ~2-fold changes. Values of the exogenous miRNA spike-in control RNA (mir-ath-159a), and of highly abundant endogenous miRNAs in the sample such as mir-486-3p, were also very similar in both groups (AD/control ratio for mir-ath-159a sequences = 1.07, for mir-486-3p = 0.94).

The Illumina platform is known to match other deep sequencing platforms generally in terms of miRNA expression levels, but with some notable exceptions for several individual miRNAs [38]. The very high expression that we observed for mir-486-5p in all samples, and the apparent very low expression of mir-124 and mir-129-5p, may be, at least in part, related to the Illumina platform [38].

All but two samples had complete blood count (CBC) data. As shown in Table 3, as expected, females had significantly lower mean values of RBC, hemoglobin and hematocrit than males, and this difference was significant in AD and control subgroups as well. In view of a prior report of anemia in AD [39], it is interesting that we found significant differences in hemoglobin and mean corpuscular hemoglobin concentration in AD males relative to control males, although this was not observed in AD females relative to control females nor in all AD samples vs. all controls (Table 3).

**Table 3:** Complete blood count (CBC) data on the samples in this study.

Group Means	WBC	RBC	Hg	Hct	MCV	MCH	MCHC	RDW	Platelets
All AD	6.17	4.57	13.68	41.5	91	30.04	33.01	14.19	224.39
All Controls	6.64	4.6	14.15	42.47	92.58	30.85	33.32	13.82	219.34
All Females	6.43	4.37	13.21	39.81	91.36	30.34	33.2	13.91	234.72
AD F	6.39	4.36	13.06	39.45	90.79	30.06	33.12	14.1	240.44
Control F	6.46	4.39	13.34	40.12	91.83	30.56	33.26	13.78	230.15
All M	6.39	4.82	14.72	44.45	92.32	30.6	33.14	14.09	207.25
AD M	5.96	4.78	14.28	43.43	91.2	30.03	32.92	14.29	209.29
Control M	6.88	4.88	15.23	45.61	93.59	31.25	33.39	13.88	204.93
**p-values:**									
All F vs All M	0.9259	**5.91E-05**	**2.09E-07**	**1.99E-07**	0.4774	0.6185	0.7328	0.4239	**0.049**
AD F vs AD M	0.4582	**0.0066**	**0.0013**	**0.001**	0.8175	0.9624	0.4159	0.596	0.1633
Con F vs Con M	0.4463	**0.0035**	**1.04E-05**	**2.81E-05**	0.3833	0.3714	0.6202	0.6994	0.1605
All AD vs. con	0.2349	0.8084	0.1429	0.3214	0.2378	0.1141	0.0935	0.0861	0.7209
AD F vs con F	0.9028	0.7906	0.3393	0.4718	0.6151	0.5319	0.6075	0.3034	0.5269
AD M vs con M	0.1235	0.5336	**0.0299**	0.109	0.1648	0.0601	**0.0481**	0.197	0.8532

Shown are the mean values for each group (AD, controls) and for males and females, both overall and within each group. Parameters which are significantly different across groups by two-tailed t-test, unpaired, at p = 0.05 or better, are shown in bold. Parameters are white blood cell count (WBC), red blood cell count (RBC), Hemoglobin (Hg), hematocrit (Hct), Mean corpuscular volume (MCV), Mean corpuscular hemoglobin (MCH), Mean corpuscular hemoglobin concentration (MCHC), Red cell distribution width (RDW), and platelet count.

### miRNA expression analyses

Because of the large number of sequences measured (and statistical tests performed), little importance should be given to any individual sequence that exhibits nominal statistical significance between groups (i.e., at p = 0.05 or better) unless they satisfy additional statistical or biological criteria. For example, an individual sequence may show a particularly large difference (e.g., >2-fold) or show very low p-value (e.g., p<0.001). Alternatively, all or most of the variant sequences aligning to a single locus may show changes in the same direction. Finally, multiple miRNAs that are biologically linked (through common biogenesis or common functions) may show changes in the same direction across groups and/or may show correlated changes across individual samples.

In preliminary studies, we examined the mature miRNA dataset manually looking for changes that might transcend individual sequences, for example, an overall increase in the occurrence of RNA editing, 5’ sequence variants, or 3’ length variability. We did not detect such global alterations, and so the analysis was carried out primarily on the level of individual miRNA loci.

#### Individual miRNA changes

We calculated the sum of all sequences aligning to each mature miRNA reference sequence in miRBase, in each sample, and screened for miRNAs that showed significant differences across groups using the Kruskal-Wallis test at p< 0.05 or better (almost all of these were also significantly different by t-test). As shown in Table 1, 20 miRNAs passed this initial screening process.

Seven of these miRNAs were also significantly different based on the individual sequence that was most abundantly expressed in the overall dataset (Table 1), of which all were lower in the AD group. Among the seven miRNAs, mir-342-3p is perhaps the most interesting, since it achieved the highest level of statistical significance by t-test, and because mir-342-5p, which is processed from the same pre-miR, was also significantly altered by a similar degree and in the same direction. Mir-342-3p also shares a 5’-seed targeting motif (TCACA) with another miRNA on the list, mir-23b-3p, as well as two other miRNAs that just missed the criteria for significance (mir-23a-3p and mir-7-1-3p). Finally, mir-342-3p is brain-enriched in its tissue expression [40]. Two other interesting pairs are mir-23b-3p and mir-24-3p, which are encoded very near each other in the genome, and mir-338-3p and mir-3065-5p, which are encoded directly opposite each other in sense/antisense fashion. These biological and statistical relationships give high confidence that the seven miRNAs are truly differentially expressed in the AD group.

Besides examining the miRNA dataset, we also screened non-miRNAs for individual sequences which showed apparent group differences based on nominal statistical significance. Sequences mapping to several rRNA, tRNA and HY1 RNA loci were observed to show down-regulation in AD, but these were not analyzed in detail, and further analysis is required to understand the biological significance of these findings. An incidental finding was that two samples (one in each group) had relatively high expression of bacterial rRNA sequences, in the face of normal miRNA levels. This cannot represent contamination of laboratory or sequencing equipment, since samples were pooled in groups of 8–12 and bar-coded. However, it is not clear whether this represents bacterial contamination of individual samples during handling, or reflects the state of the individual at the time of blood draw (e.g., having transient bacteremia or a “leaky gut”). All sequences produced in this study have been deposited in the NCBI SRA Repository.

### Analysis of co-expressed pairs and modules of miRNAs

Because miRNAs are often regulated in groups, we analyzed not only individual changes in mean miRNA expression levels, but changes in how pairs of miRNAs were correlated with each other across individual samples, both across the entire dataset as well as differentially in the AD group vs. controls. Correlation analysis is complementary to expression analysis, since two miRNAs may be highly correlated regardless of whether either one shows any mean change across groups. For this analysis, we used the nonparametric rank Spearman rho correlation coefficient, rather than the Pearson r, as being more robust.

Many pairs of miRNAs were highly (rho>0.5) correlated with each other in both groups (see S6 miRNA Pair Correlations). The largest clique (a set in which all miRNAs are significantly pairwise correlated with each other) consisted of 25 miRNAs (let7a-5p, let7c-5p, let7d-5p, let7e-5p, let7f-5p, let7g-5p, miR-126-5p, 155-5p, 199a-3p, 199b-3p, 26a-5p, 26b-5p, 30c-5p, 32-5p, 335-3p, 340-3p, 369-5p, 374a-3p, 374c-3p, 411-5p, 454-3p, 493-5p, 628-5p, 889-3p, 98-5p). Such groups of miRNAs may be correlated either because they are co-expressed together in exosomes, and/or because they are co-regulated (e.g., transcribed, processed or turned over together).

Cases in which pairs or modules of miRNAs were highly correlated in the AD group but not in controls, or vice versa, may suggest changes in the regulatory network that occur in AD. Groups of miRNAs that are highly correlated in only one group might include miRNAs that are co-expressed in a tissue which is altered across groups; co-localized in secretory exosomes whose secretion is altered across groups; or that are driven together by transcription factors which are differentially induced across groups, among other possibilities.

The miRNA pairs that exhibited large differences in the Spearman correlation values between the AD group and controls are shown in the S2 Dataset. Of these, the most statistically reliable and biologically striking findings are those in which miRNA cliques of size four or greater show high correlations in one group but not the other. One module consisting of four miRNAs (126-3p, 140-5p, 576-5p, 641) was pairwise highly correlated in the AD group but not in controls. All are intronic miRNAs, but otherwise it is not clear what they have in common. Mir-126-3p and mir-140-5p are broadly expressed in many tissues [40], but the tissue distributions of the other two are not well characterized. In contrast, a distinct clique of four miRNAs (7-1-3p, 130b-3p, 138-5p, 320b) was pairwise highly correlated in controls but not in AD samples. All four of these miRNAs are expressed abundantly or predominantly in brain. miR-138 is particularly well studied in neuroscience, and has been implicated in human memory performance [41], regulation of dendritic spines [42] and phosphorylation of tau protein [43], among other effects that may be relevant to cognition and Alzheimer disease. As well, miR-320b is a possible regulator of human-specific neural development [44]. A possible explanation for this finding is that neural exosomes containing these miRNAs are released into the circulation in control subjects (but to a lesser extent in AD subjects).

Note that mir-342-3p was highly correlated with several of the miRNAs that were down-regulated in AD (Table 1). Specifically, mir-342-3p was highly correlated with mir-342-5p, 150-5p, 23b-3p, and 29b-3p in the AD group as well as the control group (S2 Dataset). This provides additional evidence that the set of down-regulated miRNAs are not merely artifacts of screening, and suggests that the force(s) leading to down-regulation in AD affect these miRNAs as a group and not individually.

#### Use of machine learning methods to identify miRNA profiles that predict AD diagnosis

As described in Methods, machine learning was used to define seven miRNAs whose expression profile gave optimized prediction of AD vs. control status. Because the predictions were made using cross-validation, the same data were not used for training and testing and so were not subject to overfitting limitations. The best model accurately assigned, on average, 29 of 35 AD samples and 31 of 35 control samples.

To evaluate how robust these predictions are to replications of the same experiment, we trained the model on the expression values on the mature miRNA dataset, and then tested predictions on data obtained from a separate sequencing run that was carried out of the same 70 RNA samples. These technical replicate sequence data were filtered, normalized and converted to fold-changes as described for the original dataset. On average, across all miRNAs, the mean expression values for the first vs. the technical replicate datasets differed by ± 30%, and this variability was similar at both low and high miRNA expression levels (SI miRNA Expression Dataset). This amount of “noise” is consistent with other deep sequencing studies. The performance of the AdaboostM1 model on the technical replicate data (Precision: 0.848, Recall: 0.800, F1: 0.824, AUC: 0.915, MCC: 0.658) was only slightly worse than its performance on the original dataset (Table 2). The AUC, which measures the rank ordering of predictive scores regardless of the cut-off value used to discriminate AD vs. controls, was almost unchanged (0.919 vs. 0.915).

## Discussion

We demonstrated that it is methodologically feasible to prepare an enriched fraction of exosomes from human plasma and to reliably characterize expression of small RNAs by Illumina deep sequencing. We chose to study the P3 fraction, isolated by several cycles of differential centrifugation including a final ultracentrifugation step. This fraction should be much “cleaner” than exosomal RNA preps isolated using commercial exosome isolation kits; in particular, the P3 pellet should be relatively free of cell debris and microvesicles (which are likely to be removed in the P1 and P2 fractions, respectively) and soluble protein complexes (which are likely to remain in the ultrasupernatant). However, it is still possible that the P3 pellet may contain non-exosomal RNAs, either adsorbed to the surface of exosomes or present in protein complexes that were co-precipitated at high speed. It is also possible that some of the P3 pellet-associated miRNAs originated from non-exosomal miRNAs that entered circulating exosomes [45].

The AD samples were generally comparable to controls insofar as they did not exhibit differences in Complete Blood Count (CBC) parameters, global shifts in miRNA expression levels, high variability in expression levels, or a significant number of outlier samples. Screening of individual loci indicated that 20 miRNAs showed differential expression in AD, (Table 1). Notwithstanding the issues associated with multiple testing, most of these miRNAs satisfied additional criteria suggesting that the changes are biologically as well as statistically significant. Seven of the altered miRNAs were highly informative in a machine learning model for predicting the status (AD vs. controls) of individual samples. Knowing the expression levels of these 7 miRNAs alone was sufficient to predict the status of an individual sample with 83–89% accuracy. This performance is not due to over-fitting, because a) we used separate samples for training and testing, and b) similar performance was achieved when tested on technical replicate data obtained from a separate sequencing run. Also, perfect performance may not be expected since subjects were labeled “AD” or “Control” based on clinical diagnosis at the time of blood draw, rather than the gold standard of autopsy confirmation. Thus, it is likely that a few “control” subjects might have had been in subclinical stages leading to AD, and some subjects diagnosed as AD might have suffered some other form of dementia instead.

Perhaps the most interesting single miRNA was mir-342-3p, which was expressed in the AD group at about 60% of control levels. This miRNA is expressed at higher levels in brain than in most other tissues [32], and its expression was highly correlated across individuals (in both AD and controls) with several of the other miRNAs that were significantly down-regulated in AD (342-5p, 150-5p, 23b-3p, 29b-3p). It also shares a 5’-seed targeting motif with another down-regulated miRNA, mir-23b-3p, as well as two other miRNAs that just missed the criteria for significance (mir-23a-3p and mir-7-1-3p). It is noteworthy that two previous studies reported that circulating mir-342-3p is down-regulated in AD (in serum [19] and in “exosomal RNA” prepared from serum using commercial kits [15]), had high predictive value for predicting patient status in individual samples [15, 19], and correlated with Mini-Mental State Examination score [19]. Thus, the down-regulation of mir-342-3p is arguably the most robust replication of a circulating miRNA biomarker finding to date.

It is difficult to compare our findings directly to other AD biomarker studies that have studied RNAs isolated from blood fractions different from ours (whole blood, plasma, serum or “exosomal RNA” isolated by commercial kits), and moreover, studies vary in their criteria for listing a given miRNA as significantly “altered”. Nevertheless, besides mir-342-3p, several additional miRNAs that were altered in our study have also been reported to be altered, consistently in the same direction, in previous studies of circulating miRNAs in AD. For example, mir-29b-3p has been described as down-regulated in serum [11, 26], whole blood [20] and peripheral blood mononuclear cells [28]. As well, mir-125b has been described as down-regulated in serum [26, 27]. Mir-23a-3p, which just missed the cut-off of significance in our study, was significantly down-regulated in several other studies [19, 27]. In contrast, mir-15a-5p was up-regulated in plasma and in plasma-derived “exosomal RNA” [12, 15] but showed no appreciable change at all in our study.

Besides examining changes in miRNAs individually, we also looked for pairwise correlations among pairs of miRNAs, looking for pairs which were highly correlated in one group but not at all in the other. We identified a clique consisting of four miRNAs (126-3p, 140-5p, 576-5p, 641) which were pairwise all highly correlated in the AD group but not in controls, and another clique of four miRNAs (7-1-3p, 130b-3p, 138-5p, 320b) which were pairwise highly correlated in controls but not in AD samples. The reason(s) for the co-regulation of the first clique in AD are not obvious, but the second clique consists of brain-enriched miRNAs, and a possible explanation is that neural exosomes containing these miRNAs are released into the circulation in control subjects (but to a lesser extent in AD subjects).

In conclusion, the P3 fraction of plasma, isolated by differential centrifugation and enriched in exosomes, revealed a profile of miRNA changes occurring in AD. The expression of seven miRNAs was sufficient to allow highly accurate prediction of group identity in individual samples. The findings encourage further studies of exosomes that are further enriched for neural markers, which possibly may show even more selective findings [29, 46]. The findings also warrant replication and follow-up with a larger cohort of patients and controls who have been carefully characterized in terms of cognitive and imaging data, other biomarkers (e.g., CSF amyloid and tau levels), and risk factors (e.g., apoE4 status), and who are sampled repeatedly over time. Such a larger study will allow one to examine whether any miRNA alterations are specifically related to risk, onset or progression of the disease. Integrating miRNA expression data with other data is likely to provide more informative and robust biomarkers.

## Supporting Information

S1 Table Demographics and raw sequence counts for each sample. Each sample is shown according to its group identity (AD or control), sample number, gender, age at time of blood draw, and the raw sequence counts from deep sequencing (total, aligning to the human genome Hg19 accession, miRBAse hairpin miRNA database, and mature miRNA database, respectively).
(DOCX)

S1 Fig Flowchart of Eligibility Screening. This figure shows the screening process. A total of 100 eligible samples (50 AD and 50 controls) were identified. As discussed in Methods, 10 samples were used for preliminary methodological studies, and 76 were processed and analyzed in parallel to characterize changes in miRNA expression across groups. (The last arriving 14 samples were not processed due to time constraints.)
(DOCX)

S2 Fig Flowchart of Bioinformatics Pre-Processing of RNA sequences. Raw reads were trimmed to remove adaptor sequences, collapsed to create unique sequence IDs for each unique sequence across all samples, aligned to one of four reference databases, and counts for each sample were displayed in tables created separately for each of the reference databases. These were then normalized and analyzed further (not shown). Only the data from the mature miRNA miRBAse reference database were analyzed in detail in the present paper, though all data are being deposited into the NCBI SRA Repository.
(PDF)

S1 Methods Details of Bioinformatics Pre-Processing of RNA sequences. (DOCX)

S1 Dataset Filtered and normalized miRNA expression values for all sequences that aligned to the mature miRNA miRBAse reference database. Also shown for each sequence are the sum across all samples, the AD group mean, control mean, AD/Control ratio, and p-values for each sequence (group AD vs. controls) by t-test (2-tailed, unpaired, unequal variance) and by Kruskal-Wallis test (i.e., nonparametric ANOVA).
(XLSX)

S2 Dataset For each miRNA locus in the filtered and normalized dataset, we first calculated the sum of all sequences that aligned to that locus. Then, for each pair of miRNA loci, we calculated the Spearman rank correlation rho value in the AD group and separately in the Control group. The p-values indicate confidence that each rho value is significantly different from zero. Shown also is the difference in correlation values across groups (AD rho value minus Control rho value) and the absolute value of the difference. To assist in sorting the spreadsheet, we also indicate in which pairs a) AD rho and Control rho are both > 0.5; b) AD rho > 0.5 and [AD rho minus Control rho] > 0.5; c) Control rho > 0.5 and [Control rho minus AD rho] > 0.5; d) both AD and Control rho < -0.5; e) AD rho <-0.5, and [Control rho minus AD rho] > 0.5; f) Control rho < -0.5, and [AD rho minus Control rho] > 0.5.
(XLSX)
