# Supplementary material for: Plasma Exosomal miRNAs in Persons with and without Alzheimer Disease: Altered Expression and Prospects for Biomarkers
Source: PLoS One. 2015 Oct 1;10(10):e0139233. doi: 10.1371/journal.pone.0139233 (PMC4591334; doi:10.1371/journal.pone.0139233)
Supplement: S1 Table — Each sample is shown according to its group identity (AD or control), sample number, gender, age at time of blood draw, and the raw sequence counts from deep sequencing (total, aligning to the human genome Hg19 accession, miRBAse hairpin miRNA database, and mature miRNA database, respectively). (DOCX) [file pone.0139233.s001.docx]

**SI Table 1. Demographics and raw sequence counts for each sample.**

| **Group** | | **Sample** | | | | **Gender** | | **Age** | | | | **Raw Reads** | | | | | **hg19 Mapped** | | | | | **miR hairpins** | | | | **mature miRs** | | | |  |  |
| --- | --- | --- | --- | --- | --- | --- | --- | --- | --- | --- | --- | --- | --- | --- | --- | --- | --- | --- | --- | --- | --- | --- | --- | --- | --- | --- | --- | --- | --- | --- | --- |
| AD | | 22 | | | | F | | 51 | | | | 17153322 | | | | | 14082060 | | | | | 13051325 | | | | 12591184 | | | |  |  |
| AD | | 41 | | | | F | | 53 | | | | 30571545 | | | | | 26379160 | | | | | 22949199 | | | | 22362250 | | | |  |  |
| AD | | 75 | | | | F | | 59 | | | | 11817987 | | | | | 8410728 | | | | | 9027767 | | | | 8861045 | | | |  |  |
| AD | | 45 | | | | F | | 60 | | | | 680519 | | | | | 282235 | | | | | 236423 | | | | 233803 | | | |  |  |
| AD | | 27 | | | | F | | 61 | | | | 9469136 | | | | | 5878269 | | | | | 6183755 | | | | 6027870 | | | |  |  |
| AD | | 18 | | | | F | | 65 | | | | 1856400 | | | | | 1144699 | | | | | 1454096 | | | | 1427177 | | | |  |  |
| AD | | 17 | | | | F | | 66 | | | | 3657644 | | | | | 2396730 | | | | | 2508758 | | | | 2446060 | | | |  |  |
| AD | | 42 | | | | F | | 66 | | | | 11647766 | | | | | 9807056 | | | | | 10457916 | | | | 10230553 | | | |  |  |
| AD | | 83 | | | | F | | 66 | | | | 7835428 | | | | | 5764286 | | | | | 5927094 | | | | 5729061 | | | |  |  |
| AD | | 32 | | | | F | | 67 | | | | 8378867 | | | | | 5659975 | | | | | 6073533 | | | | 5875582 | | | |  |  |
| AD | | 46 | | | | F | | 67 | | | | 20223222 | | | | | 2990096 | | | | | 718827 | | | | 637121 | | | |  |  |
| AD | | 35 | | | | F | | 71 | | | | 7666963 | | | | | 4706081 | | | | | 5005720 | | | | 4811130 | | | |  |  |
| AD | | 37 | | | | F | | 72 | | | | 22335676 | | | | | 14252204 | | | | | 15225577 | | | | 14712115 | | | |  |  |
| AD | | 71 | | | | F | | 72 | | | | 14090849 | | | | | 11187233 | | | | | 10318604 | | | | 10014890 | | | |  |  |
| AD | | 36 | | | | F | | 73 | | | | 19624094 | | | | | 14525465 | | | | | 15399538 | | | | 14886970 | | | |  |  |
| AD | | 52 | | | | F | | 73 | | | | 4510814 | | | | | 1464048 | | | | | 1249001 | | | | 1233330 | | | |  |  |
| AD | | 51 | | | | F | | 74 | | | | 17605602 | | | | | 9221360 | | | | | 9226519 | | | | 9024087 | | | |  |  |
| AD | | 28 | | | | M | | 53 | | | | 15158667 | | | | | 11416046 | | | | | 10797654 | | | | 10521704 | | | |  |  |
| AD | | 64 | | | | M | | 53 | | | | 555137 | | | | | 257509 | | | | | 202292 | | | | 200088 | | | |  |  |
| AD | | 70 | | | | M | | 56 | | | | 4882406 | | | | | 2878539 | | | | | 2908770 | | | | 2829568 | | | |  |  |
| AD | | 11 | | | | M | | 57 | | | | 10746666 | | | | | 7354726 | | | | | 8905806 | | | | 8714317 | | | |  |  |
| AD | | 21 | | | | M | | 58 | | | | 19141712 | | | | | 15697602 | | | | | 13169790 | | | | 12781388 | | | |  |  |
| AD | | 23 | | | | M | | 58 | | | | 4363867 | | | | | 3156387 | | | | | 3104297 | | | | 2991438 | | | |  |  |
| AD | | 65 | | | | M | | 62 | | | | 6622333 | | | | | 5125765 | | | | | 5595244 | | | | 5475309 | | | |  |  |
| AD | | 82 | | | | M | | 62 | | | | 6658176 | | | | | 4870882 | | | | | 5107422 | | | | 4947433 | | | |  |  |
| AD | | 59 | | | | M | | 64 | | | | 7310885 | | | | | 3825905 | | | | | 4036373 | | | | 3919678 | | | |  |  |
| AD | | 13 | | | | M | | 66 | | | | 8751719 | | | | | 4598620 | | | | | 5898188 | | | | 5803467 | | | |  |  |
| AD | | 63 | | | | M | | 67 | | | | 6461874 | | | | | 4891920 | | | | | 5094485 | | | | 4963919 | | | |  |  |
| AD | | 12 | | | | M | | 68 | | | | 239752 | | | | | 135264 | | | | | 177409 | | | | 175690 | | | |  |  |
| AD | | 58 | | | | M | | 71 | | | | 4925514 | | | | | 2726717 | | | | | 2631783 | | | | 2579094 | | | |  |  |
| AD | | 69 | | | | M | | 71 | | | | 4977564 | | | | | 3692193 | | | | | 3839317 | | | | 3757665 | | | |  |  |
| AD | | 76 | | | | M | | 71 | | | | 14707106 | | | | | 10307971 | | | | | 8047988 | | | | 7894373 | | | |  |  |
| AD | | 77 | | | | M | | 72 | | | | 5201950 | | | | | 3601658 | | | | | 3840389 | | | | 3735804 | | | |  |  |
| AD | | 57 | | | | M | | 73 | | | | 5614677 | | | | | 3399343 | | | | | 3465265 | | | | 3392715 | | | |  |  |
| AD | | 81 | | | | M | | 75 | | | | 4846842 | | | | | 3088327 | | | | | 2984525 | | | | 2916598 | | | |  |  |
| Con | | 40 | | | | F | | 52 | | | | 4631255 | | | | | 2655514 | | | | | 2834997 | | | | 2705355 | | | |  |  |
| Con | | 30 | | | | F | | 53 | | | | 13972140 | | | | | 10526812 | | | | | 10227882 | | | | 10018737 | | | |  |  |
| Con | | 79 | | | | F | | 55 | | | | 3902418 | | | | | 2550644 | | | | | 2539264 | | | | 2473878 | | | |  |  |
| Con | | 56 | | | | F | | 58 | | | | 3702796 | | | | | 1884754 | | | | | 1958918 | | | | 1898660 | | | |  |  |
| Con | | 66 | | | | F | | 58 | | | | 3542448 | | | | | 2166839 | | | | | 2085235 | | | | 2031036 | | | |  |  |
| Con | | 24 | | | | F | | 59 | | | | 7603687 | | | | | 5409252 | | | | | 5470113 | | | | 5257616 | | | |  |  |
| Con | | 39 | | | | F | | 61 | | | | 41118385 | | | | | 26969611 | | | | | 25724764 | | | | 24987898 | | | |  |  |
| Con | | 34 | | | | F | | 65 | | | | 4542164 | | | | | 3277320 | | | | | 3321707 | | | | 3242199 | | | |  |  |
| Con | | 62 | | | | F | | 67 | | | | 22242611 | | | | | 13663431 | | | | | 12312025 | | | | 11996809 | | | |  |  |
| Con | | 14 | | | | F | | 68 | | | | 4147186 | | | | | 2585523 | | | | | 3057666 | | | | 3001765 | | | |  |  |
| Con | | 85 | | | | F | | 68 | | | | 10727748 | | | | | 7556925 | | | | | 7269687 | | | | 7082886 | | | |  |  |
| Con | | 15 | | | | F | | 70 | | | | 8371603 | | | | | 4715926 | | | | | 5887065 | | | | 5787589 | | | |  |  |
| Con | | 29 | | | | F | | 70 | | | | 9776351 | | | | | 7298705 | | | | | 7356480 | | | | 7223620 | | | |  |  |
| Con | | 38 | | | | F | | 70 | | | | 8569188 | | | | | 4538181 | | | | | 4893421 | | | | 4714583 | | | |  |  |
| Con | | 50 | | | | F | | 73 | | | | 3328462 | | | | | 1811338 | | | | | 1728448 | | | | 1688695 | | | |  |  |
| Con | | 86 | | | | F | | 73 | | | | 5741437 | | | | | 3527023 | | | | | 3565421 | | | | 3470880 | | | |  |  |
| Con | | 68 | | | | F | | 74 | | | | 7975543 | | | | | 6152978 | | | | | 6150571 | | | | 5975482 | | | |  |  |
| Con | | 80 | | | | F | | 74 | | | | 10243022 | | | | | 7548284 | | | | | 7167941 | | | | 6967065 | | | |  |  |
| Con | | 54 | | | | F | | 75 | | | | 2974247 | | | | | 1180403 | | | | | 1051986 | | | | 1025605 | | | |  |  |
| Con | | 84 | | | | F | | 75 | | | | 11036073 | | | | | 7511712 | | | | | 7651432 | | | | 7465307 | | | |  |  |
| Con | | 26 | | | | M | | 50 | | | | 5639840 | | | | | 4312466 | | | | | 4523710 | | | | 4366523 | | | |  |  |
| Con | | 67 | | | | M | | 55 | | | | 18181343 | | | | | 15268266 | | | | | 14964097 | | | | 14441407 | | | |  |  |
| Con | | 16 | | | | M | | 59 | | | | 27492424 | | | | | 4898811 | | | | | 5937085 | | | | 5824049 | | | |  |  |
| Con | | 25 | | | | M | | 59 | | | | 6931496 | | | | | 4891429 | | | | | 5239624 | | | | 5111812 | | | |  |  |
| Con | | 33 | | | | M | | 60 | | | | 1471176 | | | | | 875689 | | | | | 905243 | | | | 871325 | | | |  |  |
| Con | | 55 | | | | M | | 60 | | | | 3522975 | | | | | 1235050 | | | | | 1173636 | | | | 1138484 | | | |  |  |
| Con | | 74 | | | | M | | 60 | | | | 14444632 | | | | | 11499784 | | | | | 9730238 | | | | 9397592 | | | |  |  |
| Con | | 78 | | | | M | | 62 | | | | 12154344 | | | | | 9462368 | | | | | 9850177 | | | | 9595138 | | | |  |  |
| Con | | 20 | | | | M | | 66 | | | | 1304226 | | | | | 491982 | | | | | 544995 | | | | 537921 | | | |  |  |
| Con | | 73 | | | | M | | 67 | | | | 10029745 | | | | | 7559259 | | | | | 6310135 | | | | 6126135 | | | |  |  |
| Con | | 19 | | | | M | | 68 | | | | 2182684 | | | | | 1222796 | | | | | 1533690 | | | | 1512502 | | | |  |  |
| Con | | 72 | | | | M | | 68 | | | | 8168626 | | | | | 6219616 | | | | | 6376042 | | | | 6222613 | | | |  |  |
| Con | | 49 | | | | M | | 69 | | | | 2940855 | | | | | 1785395 | | | | | 1732282 | | | | 1695012 | | | |  |  |
| Con | | 60 | | | | M | | 73 | | | | 5493960 | | | | | 3084924 | | | | | 2999993 | | | | 2906441 | | | |  |  |
| Con | | 61 | | | | M | | 73 | | | | 29057565 | | | | | 21522877 | | | | | 21060691 | | | | 20546372 | | | |  |  |
|  | |  | | | |  | | |  | | | |  | |  | | | |  | | | |  | | | |  | | | |  |
| AD mean | | |  | |  | | 64.94 | | | | 9722648.03 | | | | | 6547915.97 | | | | | 6423447.11 | | | | 6248699.31 | | | |  |  |  |
| Control mean | | | | |  | | 64.77 | | | | 9633275.86 | | | | | 6224625.34 | | | | | 6146761.74 | | | | 5980256.89 | | | |  |  |  |
| AD/Control ratio | | | | |  | | 1.00 | | | | 1.01 | | | | | 1.05 | | | | | 1.05 | | | | 1.04 | | | |  |  |  |
| t-test |  | | | |  | | 0.92 | | | | 0.96 | | | | | 0.81 | | | | | 0.83 | | | | 0.83 | | | |  |  |  |
|  | |  | |  | | | | | |  | | | |  | | | |  | |  | | | |  | | | |  | | | |

Each sample is shown according to its group identity (AD or control), sample number, gender, age at time of blood draw, and the raw sequence counts from deep sequencing (total, aligning to the human genome Hg19 accession, miRBAse hairpin miRNA database, and mature miRNA database, respectively).
