# Supplementary material for: Plasma Exosomal miRNAs in Persons with and without Alzheimer Disease: Altered Expression and Prospects for Biomarkers
Source: PLoS One. 2015 Oct 1;10(10):e0139233. doi: 10.1371/journal.pone.0139233 (PMC4591334; doi:10.1371/journal.pone.0139233)
Supplement: S1 Fig — This figure shows the screening process. A total of 100 eligible samples (50 AD and 50 controls) were identified. As discussed in Methods, 10 samples were used for preliminary methodological studies, and 76 were processed and analyzed in parallel to characterize changes in miRNA expression across groups. (The last arriving 14 samples were not processed due to time constraints.) (DOCX) [file pone.0139233.s002.docx]

535 Individuals assessed for eligibility

50 Group A (AD)

50 Group B (Healthy Controls)

By Source:

46 Clinic

4 Community

By Source:

41 Community

9 Clinic

100 Matched Samples Provided

391 Excluded

203 Did not meet basic study criteria

56 Out of geographical area

51 Out of age range

39 Other diagnosis

42 Other (e.g., currently in a study, living in a nursing home, MMSE out of range, no study partner, non-stable medical condition, on excluded medication, significant hearing/vision/language deficit, temporarily ineligible)

144 Eligible (with 44 without a match)
