# Supplementary figures and images for: Plasma Exosomal miRNAs in Persons with and without Alzheimer Disease: Altered Expression and Prospects for Biomarkers
Source: PLoS One. 2015 Oct 1;10(10):e0139233. doi: 10.1371/journal.pone.0139233 (PMC4591334; doi:10.1371/journal.pone.0139233)

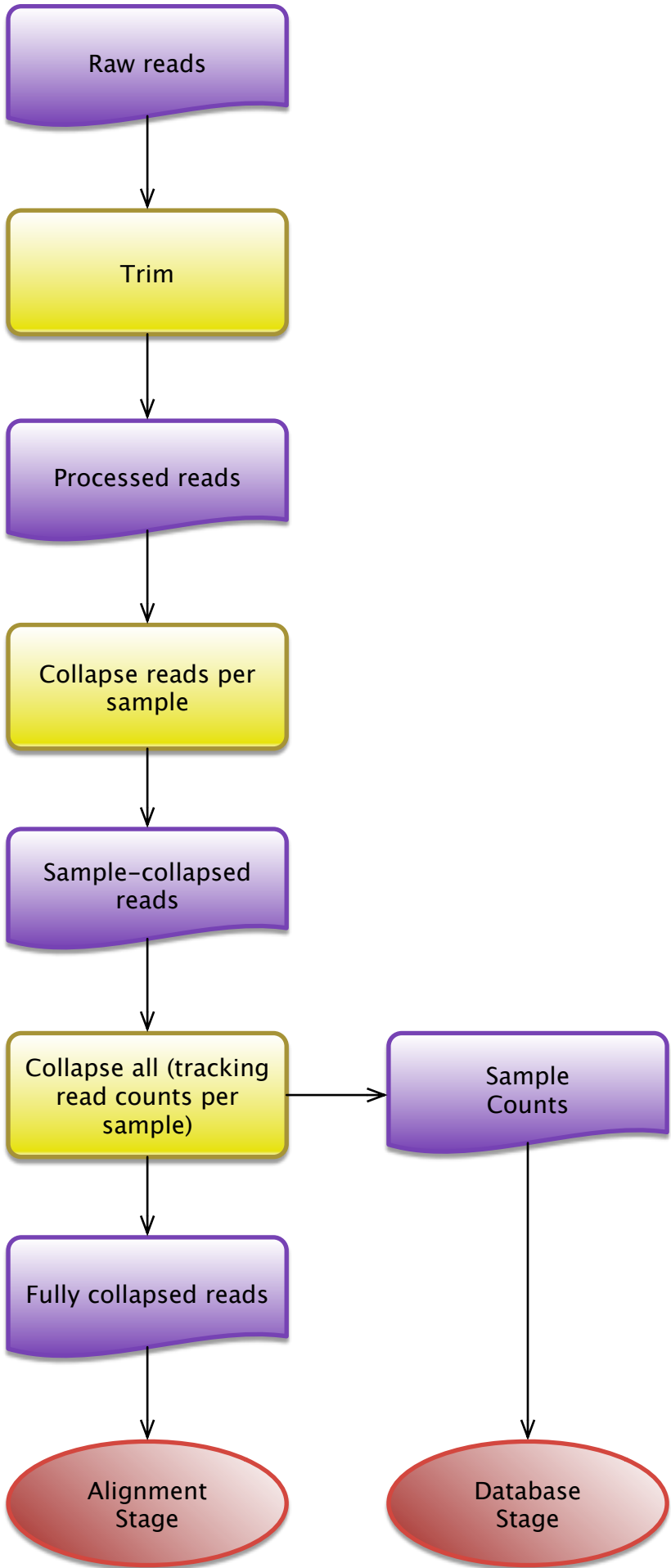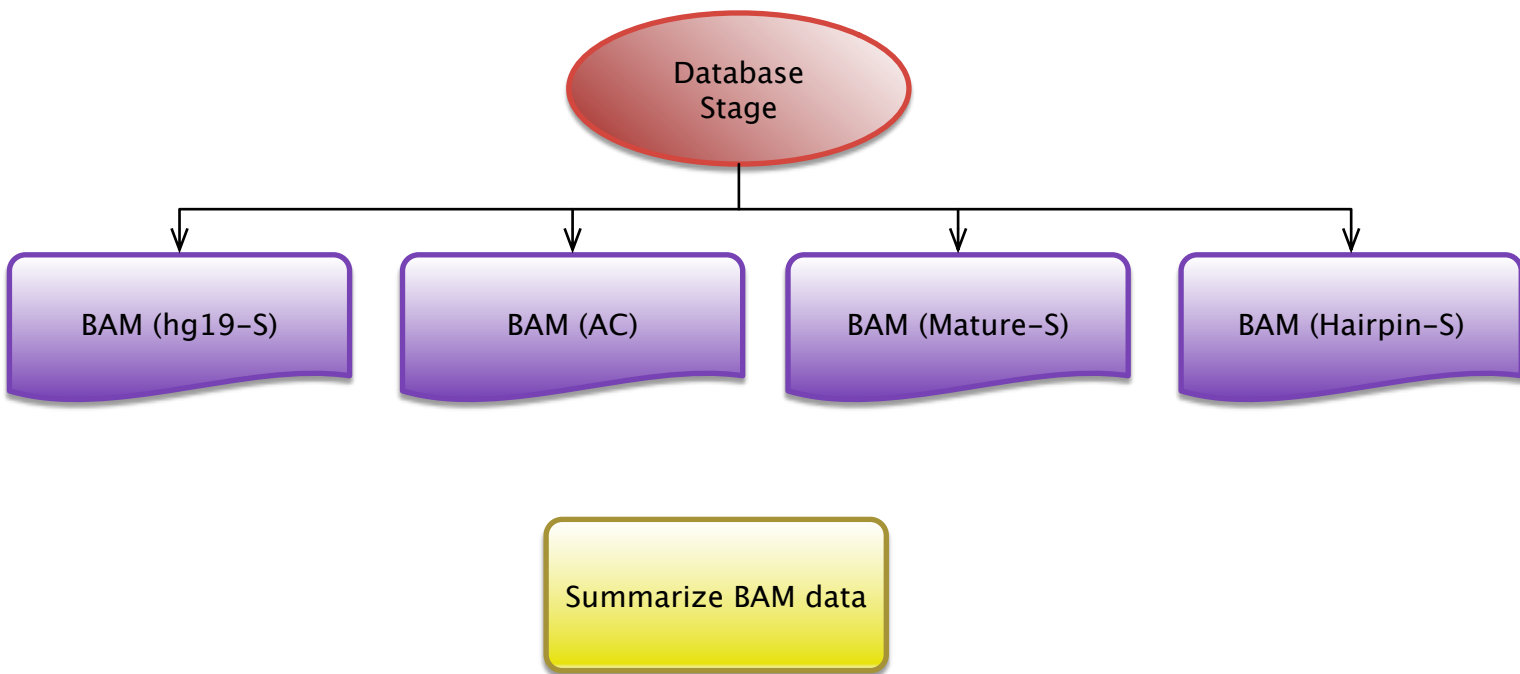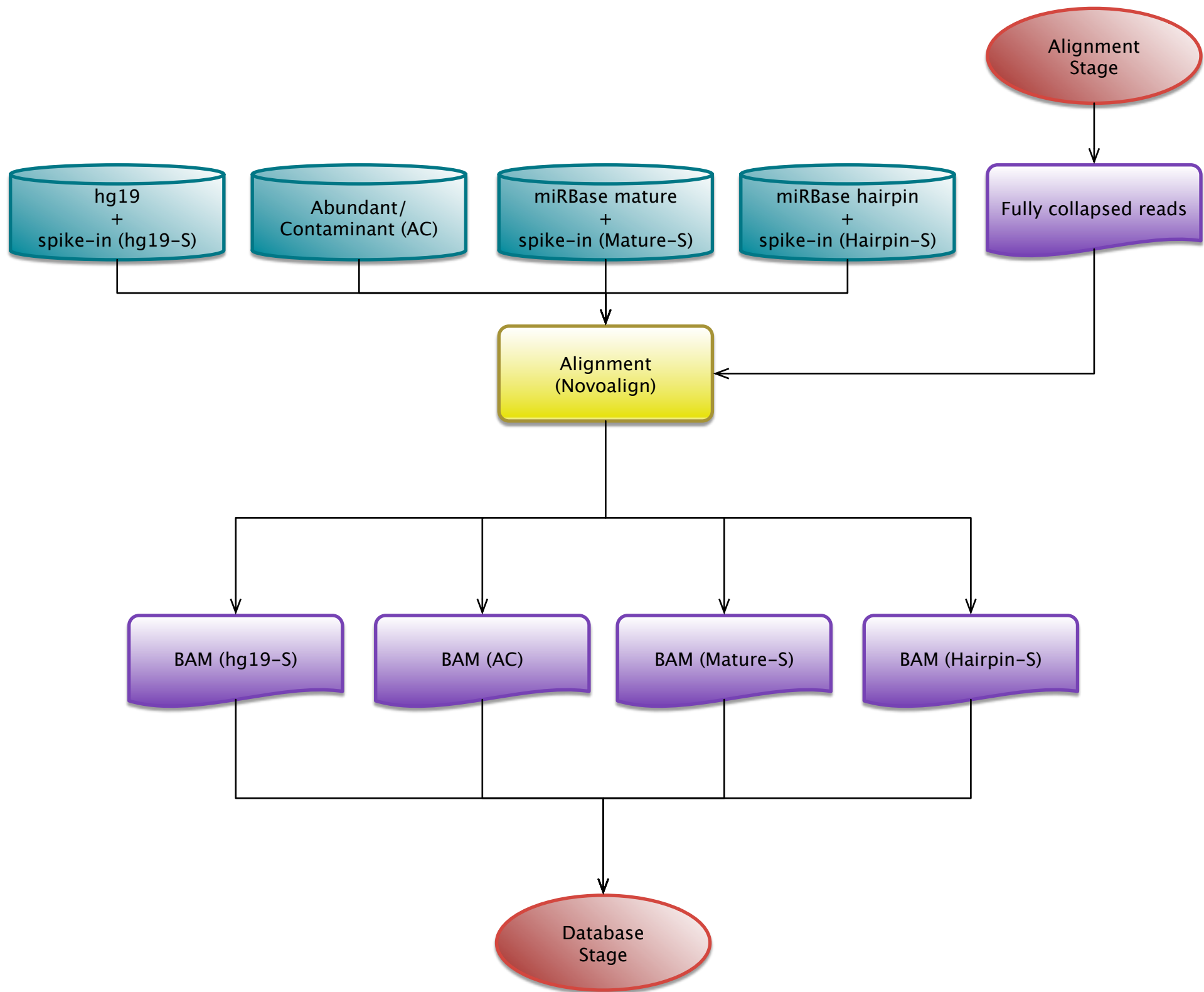

Supplement: S2 Fig — Raw reads were trimmed to remove adaptor sequences, collapsed to create unique sequence IDs for each unique sequence across all samples, aligned to one of four reference databases, and counts for each sample were displayed in tables created separately for each of the reference databases. These were then normalized and analyzed further (not shown). Only the data from the mature miRNA miRBAse reference database were analyzed in detail in the present paper, though all data are being deposited into the NCBI SRA Repository. (PDF) [file pone.0139233.s003.pdf]
