# Supplementary material for: Plasma Exosomal miRNAs in Persons with and without Alzheimer Disease: Altered Expression and Prospects for Biomarkers
Source: PLoS One. 2015 Oct 1;10(10):e0139233. doi: 10.1371/journal.pone.0139233 (PMC4591334; doi:10.1371/journal.pone.0139233)
Supplement: S1 Methods — (DOCX) [file pone.0139233.s004.docx]

# Materials and Methods

The following steps were performed using a set of Bash and Perl code available at [<https://github.com/HPCBio/miRNA-Sample-Tracker>].

## Trimming

Raw FASTQ data was adapter and quality trimmed using Trimmomatic v0.30 (Bolger, Lohse et al. 2014) using single-end mode (TrimmomaticSE) and the following parameters:

-threads 8 -phred33 -trimlog <LOGFILE> <FASTQ> ILLUMINACLIP:adaptor.fa:2:30:3 LEADING:30 TRAILING:30 MINLEN:15

The input adaptor sequence used for trimming in this case was the TruSeq Small RNA adapter, with the sequence ‘TGGAATTCTCGGGTGCCAAGGAACTCCAGTCAC’. No other adapter sequences were used for trimming, primarily to determine the levels of background adapters potentially present in samples; these (as well as homopolymeric sequences and other possibly abundant sequences) were detected downstream by aligning all sequences against an ‘abundant-contaminant’ database containing common Illumina adapter sequence along with other contaminant or abundant sequences (described below).

## Read consolidation

After trimming, redundant sequences were removed using fastx-collapser from the FASTX Toolkit v. 0.0.14 (http://hannonlab.cshl.edu/fastx_toolkit/index.html), run in a two-step process. First, each sample had redundant reads (those matching 100% identity and length) removed, retaining the abundance of the reads in each sample; these were converted from FASTQ to FASTA at this point. Second, all collapsed reads from all samples were further collapsed into one single read file. All read counts per sample were retained for downstream analyses.

Basic sample IDs and read abundance counts per sample were retained in a simple SQLite database used for downstream analysis, primarily in order to regain accurate counts due to read collapsing. These counts were validated against the original data in test runs prior to running on the full pipeline by comparing the total number of mapped and unmapped reads to the total read counts for each sample after trimming. In general, this procedure resulted in a 20-fold reduction in sequence data and dramatically simplified alignments, with the slight additional cost of sample tracking.

For simplicity the sample meta-data was retained in a separate tab-delimited text file and incorporated in downstream summary files when needed.

## Alignment

Collapsed read data were aligned using Novoalign v. V3.02.00 (http://www.novocraft.com) and the following alignment parameters:

novoalign -c 7 -d <DATABASE INDEX> -f <FASTA> -l 15 -r ALL -o SAM –m

The sequences were aligned to the following databases:

- mirBase release 20 (Kozomara and Griffiths-Jones 2014) hairpin data (human only) + ath-miR159a (spike-in mature sequence from the same miRBase release), converted from RNA to DNA,
- mirBase release 20 mature sequences (human only) + ath-MIR159a (spike-in hairpin sequence from the same miRBase release) , converted from RNA to DNA
- Human reference genome, UCSC hg19 (retrieved from iGenomes [REF]) + the ath-miR159a mature sequence from miRBase release 20
- An ‘abundant contaminant ‘ database - consists of sequences obtained from the iGenomes release for UCSC hg19 (obtained 02/27/14, http://support.illumina.com/sequencing/sequencing_software/igenome.html), which includes the standard Illumina sequence adapter, phiX174, polyA, polyC, human 5S, and human ribosomal repeating unit. In order to detect presence of other potential contaminating sequences from library preparation, the following Illumina sequences were also added: TruSeq small RNA adapters (both RA5 and RA3), the stop oligo (STP), RT Primer (RTP), and PCR primer (RP1, RPI_5, RPI_3).

Aligned data were sorted in both name and coordinate order for post-processing and data summarization. After post-alignment sorting, the SQLite database was further loaded with additional basic information from the alignments used for downstream summarization and filtering, including the databases particular unique sequences mapped to and the frequency of mapping.

## Alignment summaries

For the abundant/contaminant and miRBase alignments, simple summary files were generated containing relevant matches of sequence reads to sequences in each database; this was performed using samtools v0.1.19 (Li, Handsaker et al. 2009).

For all whole genome alignment data, a comprehensive set of features for analysis (in GTF format) was created as follows:

### sno/miRNA

Data were retrieved from the UCSC Genome Database for human genome reference using a direct MySQL connection and the UCSC command-line tools bedToGenePred and genePredToGtf, using the following UNIX commands:

mysql --user=genome --host=genome-mysql.cse.ucsc.edu -A -N \

-e "select chrom,chromStart,chromEnd,name,score,strand,thickStart,thickEnd from wgRna;" hg19 | \

bedToGenePred stdin stdout | genePredToGtf file stdin wgRna.gtf

The GTF-formatted output was sorted using BEDTools v2.20.1:

sortBed -i wgRna.gtf

Column 2 in the GTF file (the source) for these files was replaced by ‘wgRNA’ to distinguish these features in downstream analyses.

### tRNA

Data were retrieved from the UCSC Genome Database similar to that used for sno/miRNA above, using the following UNIX commands:

mysql --user=genome --host=genome-mysql.cse.ucsc.edu -A -N \

-e "select chrom,chromStart,chromEnd,name,score,strand from tRNAs;" hg19 \

| bedToGenePred stdin stdout | genePredToGtf file stdin tRNA.gtf

This was further processed to a file containing only the exonic regions:

grep -P "\texon\t" tRNA.gtf > tRNA_clean.gtf

The source column was replaced with ‘hg19_tRNAs’ for distinugishing these features from others in the consolidated features file.

### lincRNA

lincRNA data was retrieved directly from the UCSC Table Browser due to problems with schema differences in the UCSC MySQL tables. The data were sorted (as above for sno/miRNA). The original source column ID of ‘hg19_lincRNAsTranscripts’ was retained.

As these also have similar ‘split’ gene problems outlined above for the canonical genic regions, we follow the same protocol for collapsing the split features to 5’ and 3’ coordinates, pull out the specific ‘gene’ features for that table, then sort them.

### Canonical genes

Using the genes,gtf annotation data provided with the iGenomes release 9which contains RefSeq IDs), we used the joined tables ‘knownCanonical’ and ‘kgXref’ retrieved from the UCSC genome browser and retrieved a set of canonical transcripts with their original RefSeq identifiers. The ‘knownCanonical_kgXref’ file below is the raw UCSC table output:

perl canonicalize-gtf.pl -f genes.gtf -k knownCanonical_kgXref

The source IDs for this set of data were labeled as ‘refseq_canon’, and the resulting file named ‘genes_canonical.gtf’.

For these analyses we were mainly interested in whether a particular sequence was within the 5’ and 3’ bounds for the canonical transcripts. As UCSC GTF format only contains transcript coordinates in the form of ‘split’ features (CDS or exons), we parsed though these files using the script ‘collapse-gtf.pl’ (included in source code mentioned above), creating a new simple GTF file with gene regions describing the 5’ and 3’ ends of the transcripts:

perl collapse-gtf.pl -f genes_canonical.gtf -s refseq_all > \

genes_canonical_collapsed.gtf

This file will produce both gene and transcript features. These were further filtered for only ‘gene’ feature types for downstream analyses:

grep -P '\tgene\t' genes_canonical_collapsed.gtf > \

genes_canonical_collapsed_onlygenes.gtf

Once all file were generated, the various GTF files were combined and sorted:

cat genes_canonical_collapsed_sorted_onlygenes.gtf wgRna_new.gtf \

lincRNA_collapsed_onlygenes.gtf tRNA.gtf | sortBed -i stdin > all.gtf

References

Bolger, A. M., M. Lohse and B. Usadel (2014). "Trimmomatic: a flexible trimmer for Illumina sequence data." Bioinformatics **30**(15): 2114-2120.

Kozomara, A. and S. Griffiths-Jones (2014). "miRBase: annotating high confidence microRNAs using deep sequencing data." Nucleic Acids Res **42**(Database issue): D68-73.

Li, H., B. Handsaker, A. Wysoker, T. Fennell, J. Ruan, N. Homer, G. Marth, G. Abecasis, R. Durbin and S. Genome Project Data Processing (2009). "The Sequence Alignment/Map format and SAMtools." Bioinformatics **25**(16): 2078-2079.
